# Permeability of Concrete with Recycled Concrete Aggregate and Pozzolanic Materials under Stress

**DOI:** 10.3390/ma9040252

**Published:** 2016-03-30

**Authors:** Hailong Wang, Xiaoyan Sun, Junjie Wang, Paulo J.M. Monteiro

**Affiliations:** 1 Department of Civil Engineering, Zhejiang University, Hangzhou 310058, China; hlwang@zju.edu.cn (H.W.); jjcc1201@gmail.com (J.W.); 2 Department of Civil and Environmental Engineering, University of California Berkeley, Berkeley, CA 94720, USA; monteiro@berkeley.edu

**Keywords:** chloride transportation, recycled aggregate concrete, compressive stress, flexural stress, pozzolanic additive

## Abstract

The research reported herein studied the permeability of concrete containing recycled-concrete aggregate (RA), superfine phosphorous slag (PHS), and ground granulated blast-furnace slag (GGBS) with and without stress. Test results showed that the chloride diffusion coefficient of RA concrete (RAC) without external loads decreased with time, and the permeability of RAC is much lower than that of the reference concrete due to the on-going hydration and the pozzolanic reaction provided by the PHS and GGBS additives in the RAC mixture. The permeability of chloride under flexural load is much more sensitive than that under compressive load due to the differences in porosity and cracking pattern. At low compressive stress levels, the permeability of chloride decreased by the closing of pores and microcracks within RAC specimens. However, in a relatively short time the chloride diffusion coefficient and the chloride content increased rapidly with the increase of compressive stress when it exceeded a threshold stress level of approximate 35% of the ultimate compressive strength. Under flexural stress, the chloride transport capability increased with the increase of stress level and time. At high compressive and flexural stress levels, creep had a significant effect on the permeability of chloride in the RAC specimens due to the damage from the nucleation and propagation of microcracks over time. It is apparent that mortar cracking has more of a significant effect on the chloride transport in concrete than cracking in the interfacial transition zone (ITZ).

## 1. Introduction

Recycling discarded concrete as new concrete aggregate has been proven to be a commercial and effective way for the sustainable development. However, the recycled-concrete aggregate (RA) is composed of adhered mortar and original aggregate, meaning the internal defects and the adhesive mortar on the surface of RA cause the physical and mechanical properties of recycled aggregate concrete to deteriorate. Compared with the performance of natural aggregate concrete (NAC), the shrinkage and the water sorptivity of RA concrete (RAC) increased, but the strength, the elastic modulus and the durability decreased, which limits the application of RAC in structural concrete [1,2,3].

Various methods have been suggested to compensate for the shortcomings in RAC, and incorporating pozzolanic materials have been accepted as effective ways to improve the performance of RAC [4,5]. Our previous study showed that mixing phosphorous slag (PHS) and ground granulated blast-furnace slag (GGBS) in concrete can enhance its mechanical properties, especially the long-term mechanical properties of RA concrete [6]. The compressive strengths and the elastic modulus of RAC containing 25% recycled aggregate, 10% PHS and 10% GGBS are higher than those of the reference nature aggregate concrete. Just from the mechanical index, this mixture of RAC can be used as structural concrete. However, when the reinforced concrete works in the marine environment, the chloride transportation is a most important factor determining the corrosion and the service life of reinforced RAC structures. The RAC with good mechanical properties should also have a good resistance to the chloride penetration. Therefore, one purpose of this study is to identify the permeability of RAC with PHS and GGBS materials before applying it to marine structures.

During their service life, concrete structures are always subjected to various types of loads and aggressive environments, so it is essential to evaluate the transport properties of RAC under a simultaneous application of stress [7,8]. Nowadays, the ingress of nocuous compounds into unstressed concrete has been studied in great detail, but more investigations are still required to answer the relationship between stress and permeability. Several experimental studies on the water and chloride permeability of stressed normal concrete have shown that the applied loads can promote the growth of microcracks and interconnectivity, which in turn results in an increase of permeability of structural concrete [7,9,10,11,12,13]. It seems that the design service life of concrete structures will be overestimated if this effect is not taken into account. It is important to understand the influences of combined mechanical and environmental loads on the durability of recycled aggregate concrete. However, very little is known about this issue. Furthermore, the microstructure of RAC is more complicate, and therefore the conclusions drawn from common concrete might not be applied to the RAC evaluation. Therefore, another purpose of this experimental study is to address the influences of applied loads on the chloride transport in unmacrocracked but stressed RAC.

Experimental studies also show that the chloride diffusion coefficient of common concrete often decreases with time due to the further hydration of concrete. For stressed concrete, there are more time-dependent factors, such as creep, microstructure and hydration. These factors influence the permeability of stressed concrete, but they are seldom discussed in the previous studies. This study used the natural diffusion method to evaluate the chloride permeability of RAC. Therefore, the effect of creep or time on the chloride transport in RAC will be revealed to better understand the RAC.

## 2. Experimental Program

### 2.1. Materials

The cement was a 42.5R ordinary Portland cement produced in Hangzhou, China, that complied with the Chinese National Standard. The fine aggregate used in the mix was a local river sand with a fineness modulus of 2.60. The natural coarse aggregate (NA) was a locally available crushed gravel with a maximum size of 20 mm. The apparent density of NA was 2610 kg/m^3^, and the crushing index was 7.3%. The recycled-concrete aggregate (RA) was obtained from the demolition debris of old concrete pavement in Hangzhou by mechanical crushing. The average compressive strength of the concrete cores extracted from the original pavement was 30 MPa. The particle sizes of RA used in this mixture graded from 5 to 20 mm with a crushing index of 13.8%. This recycled concrete aggregate had a water absorption capacity of 3.63% by weight and an apparent density of 2539 kg/m^3^. The recycled fine aggregate (particle size <5 mm) was not used in the mixture due to its even higher absorption capacity and higher shrinkage characteristic. 

Superfine phosphorous slag (PHS) with a Blaine surface area of 350 m^2^/kg, which was a by-product from the manufacture of yellow phosphorus via electrical furnace method in Guizhou, China, was added to the mixtures. The PHS has similar chemical components to blast furnace slag, whose chemical composition (in percent weight) was listed in Table 1. The ground granulated blast-furnace slag (GGBS) employed in this study was produced in Shandong, China and had a Blaine fineness of 516 m^2^/kg (Grade S105) with a density of 2.91 g/cm^3^; it also complied with the Chinese National Standard. The chemical composition of GGBS was displayed in Table 1, in which the total glass content was 89%. To reduce the slump loss caused by superfine pozzolanic powders and RA, a superplasticizer (naphthalin-type) was added into the RAC mix. The amount was 1.5% by the mass of the cementitious materials. The mixing water in this experiment was local tap water.

Referring to the previous report [6], the concrete containing 25% RA (as replacement of NA by mass), 10% PHS and 10% GGBS (as replacement of cement by mass) has the best performance in the ten group mixtures, whose mechanical properties are even higher than those of the reference nature aggregate concrete (NAC). Therefore, this kind of RAC and the reference NAC were adopted in this experiment, and their mix proportions are listed in Table 2. To achieve a similar workability with NAC, a four-stage mixing method was adopted to produce the RAC [6]. Firstly, RA, NA, mineral admixtures and superplasticizer were mixed together for 30 s. After that, 20% of total water was added into the mix and blended for 30 s. Then, cement and sand were added and mixed for another 30 s. Finally, the remaining mixing water was poured into the mix and blended for 60 s.

### 2.2. Specimens with External Loads

**Figure 1:**
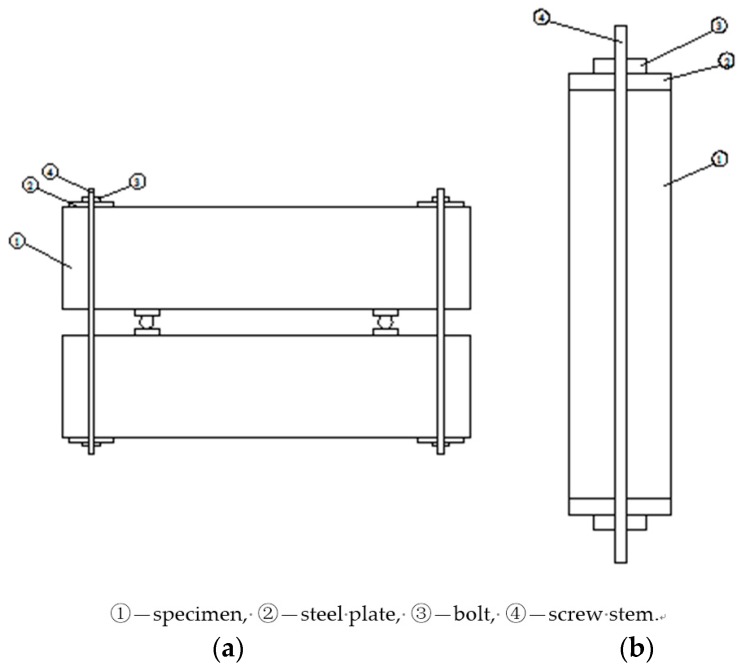
Prisms under sustained external loads: (**a**) flexural stress; (**b**) compressive stress.

Prismatic 150 × 150 × 600 mm^3^ RAC specimens were cast in a standard laboratory at 25 ± 2 °C. For every prism, a hollow cylindrical duct at suitable place was precast for applying different levels of stress to the specimens. With the exception of one test surface, all of the other five surfaces of the specimen were coated with epoxy to ensure one-dimensional chloride ingress after 28 days of curing. The methods for applying flexural and compressive loads on RAC are shown in Figure 1. The applied stresses were monitored and controlled through the attached strain gauges on the steel and concrete surfaces. If the strain did not change once screwing was done, the applied stress was satisfied with the design requirement. To reduce the relaxation caused by stress, low relaxation Q550 steel was used to form the loading system. If the stress reduction exceeded 5% of the applied stress, the nuts were screwed down until the stress returned to the intended level. All of the steel plates, screw stems, and bolts were galvanized and coated with epoxy to avoid the corrosion of steel before the experiments. Because it proved difficult to apply a higher stress level on the specimen for a long time without collapsing it, the prismatic specimens were not macro-cracked and subjected to stress ratios of 0%, 20%, 30%, 40%, 50%, 60% and 70% of their flexural and compressive strengths.

### 2.3. Experimental Environment

All specimens, including the prisms under flexural and compressive stresses and the control specimens without loads, suffered a wet-dry cycling environment. Each cycle included one week of immersion in a solution with 5% weight NaCl at 20 ± 2 °C followed by one week of drying in a tank. The experimental solution was replaced every month.

### 2.4. Chloride Transport Test

The chloride profiles in the specimens were analyzed by grinding concrete into powder from the second month through the sixth month of exposure. To evaluate the chloride migration, 20 mm diameter cores were drilled from the specimen along the central line perpendicular to the test surface. The cores were cut into slices with 5 mm depth intervals and ground into powder. To provide sufficient powder and to remove the possible discontinuity of the chloride profile that may be encountered in a specific core sample, six identical core samples were obtained for chloride profile analysis of each test series. Powders obtained in each test case were mixed together. In total, the chloride contents in the 550 powder samples were measured with acid soluble method.

It can be assumed that the predominant ionic transport process in RAC is diffusion, and this transport remains constant with time. Given this, the numerical solution to Fick’s second law can be used to calculate the chloride ion diffusion coefficients. The apparent diffusion coefficient was obtained by curve-fitting of the chloride profiles with Equation (1).

(1) $$\frac{C_{x}-C_{b}}{C_{s}-C_{b}}=1-erf(\frac{x}{2\sqrt{D_{a}t}}),$$

where *C_x_* is the chloride concentration at depth *x* and time *t*, %; *Cs* is the chloride concentration at the surface, %; *C_b_* is the initial chloride content in concrete (which can be assumed as zero in this study); and *D_a_* is the apparent diffusion coefficient in cm^2^/s.

### 2.5. Air Permeability Test

The air permeability of concrete was measured using an Autoclam permeability system. In the test, the relative pressure inside the apparatus was increased to slightly above 0.5 bar, and then the decay in pressure was recorded every minute for 15 min. The air permeability index is calculated from the absolute slope of the linear regression curve between the 5th and 15th min.

## 3. Results and Discussion

### 3.1. Permeability of Concrete without Stress

The chloride permeability and the air permeability of concretes without stress were tested, and the results are listed in Table 3. The test results in the reference [6] showed that the chloride diffusion coefficient of a concrete with 25% RA (as replacement of NA by mass) and 20% PHS (as replacement of cement by mass) was reduced to 39.3% of a concrete with 25% RA and no supplementary cementitious materials, and the chloride diffusion coefficient of a concrete with 100% RA and 20% PHS was decreased by 36.6% respected to a concrete with 100% RA and no supplementary cementitious materials. Therefore, it can be found from Table 3 and the reference [6] that the addition of PHS and GGBS decreases the permeability of RAC significantly. The chloride permeability of RAC decreased by almost 50% of the reference NAC, while the air permeability of RAC is reduced to 35% of the NAC. The SEM images in our previous study show that the pore size and the pore distribution of RAC are greatly refined due to the pozzolanic reaction of PHS and GGBS, which blocks the permeability path [6].

**Figure 2:**
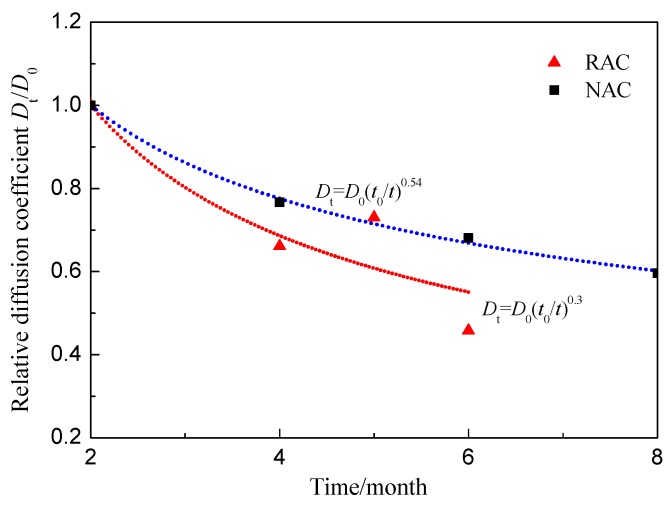
Chloride diffusion coefficients of concretes without stress after different exposure times.

Figure 2 displays the relationship between the chloride diffusion coefficient and time. It can be seen from Figure 2 that the following exponential function can be used to describe the time-dependent relationship:

*D*_t_ = *D*_0_(*t*_0_/*t*)^m^, (2)

where *D*_t_ is the diffusion coefficient at time *t*; *D*_0_ is the diffusion coefficient at reference time *t*_0_; and *m* is the age factor (0.3 for RAC and 0.54 for NAC).

As shown in Figure 2, the chloride permeability of RAC decreases more quickly than that of NAC due to the further hydration and the pozzolanic activity. The hydration process in RAC is long-lasting because of the retardation of hydration caused by the addition of PHS. The latent hydration characteristic of pozzolanic materials made a dense, insoluble calcium silicate hydrate (C–S–H). In addition, chloroaluminates formed by the reacting of chloride ions with tricalcium aluminate hydration products may reduce the porosity of concrete [14]. The formation of a protective layer of aragonite and brucite on the concrete surface can also decrease the chloride penetration. This effect is particularly relevant in the submersed and tidal zone where concrete is in contact with sea water. Compared with the mechanical and durability properties of NAC, the RAC containing 25% recycled aggregate, 10% PHS and 10% GGBS satisfies the performance requirements of structural concrete. Thereafter, for application purposes, this kind of concrete was used to identify the influence of mechanical stresses on the chloride permeability.

### 3.2. Chloride Permeability of RAC under Compressive Stress

#### 3.2.1. Chloride Profile

**Figure 3:**
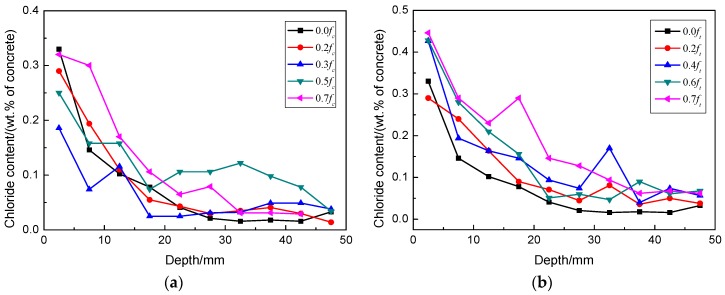
Chloride profile in RACs after 6 months’ exposure: (**a**) subjected to compressive stresses; (**b**) subjected to flexural stresses.

The chloride profile is an important parameter in predicting the service life of a structure and scheduling maintenance efforts. The chloride profiles expressed as a percentage of total chlorides by mass of RAC under external compressive stresses and the profiles subjected to no stress are shown in Figure 3a.

**Figure 4:**
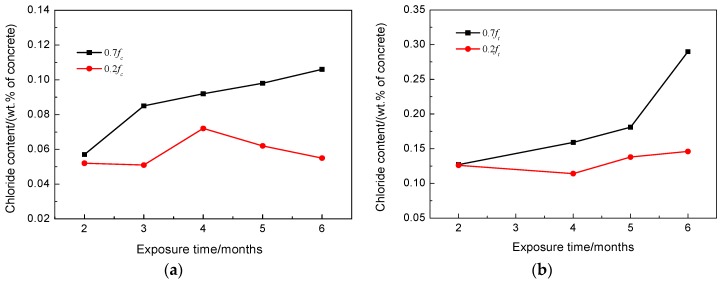
Chloride concentrations at 17.5 mm depth of concrete after different exposure times: (**a**) subjected to compressive stresses; (**b**) subjected to flexural stresses.

It can be seen in Figure 3a that the applied compressive stress levels have different effects on the chloride transport in RAC. The measured chloride concentrations at the applied compressive stresses of 0.2 and 0.3 *f_C_* (ultimate compressive strength of concrete) decreased at the same depth of RAC compared to the specimens without stress. However, the chloride content increased at a stress level of 0.4 *f_C_* and above. The chloride concentrations in RAC under compression after different exposure times are displayed in Figure 4a. The figure shows that the chloride content inside of RAC at the same depth under low stress levels increased marginally over time. However, under a high level of compressive stress, the chloride content at the same depth obviously increased over time.

**Figure 5:**
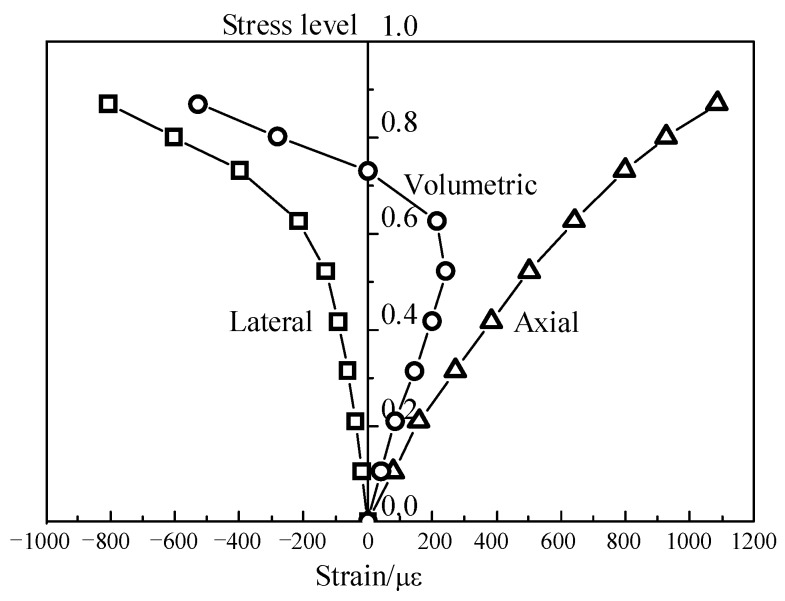
Typical stress-strain curves for RAC under uniaxial compressive load.

It is well known that the transportation of chloride into concrete is mainly dependent upon the porosity and the connectivity of the pores in concrete [8,15]. Under mechanical loads, the porosity and the connectivity strongly relate to the load type and the load level. The axial and lateral strains of RAC under uniaxial compression were recorded in the test. The typical curves are presented in Figure 5. As can be seen, the stress-strain relationship of RAC is linear elastic at relative low loads (≤0.2 *f*_c_), meaning that the microcracks in RAC are not propagated. At this stage, some microcracks, channels, and even capillary pores were closed, resulting in a volumetric contraction as shown in Figure 5, and then the reduction of porosity and chloride ingression. Thereafter, with the increase of compressive stresses, all of the stress-strain curves deviate from linearity. This indicates that more and more cracks satisfying the cracking criterion will develop and coalesce [16], which enhanced the porosity and the connectivity of pores, thus causing an increase in chloride content.

#### 3.2.2. Chloride Diffusion Coefficient

**Figure 6:**
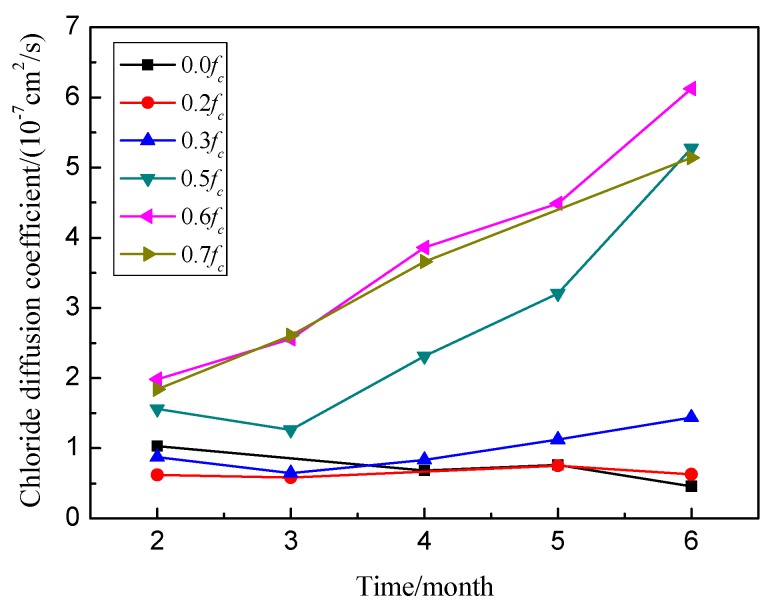
Relationship between chloride diffusion coefficients and exposure times under compressive stresses.

The diffusion coefficients *D_ac_* were obtained from chloride profiles in various exposure periods and stress levels using Equation (1). The average values of these parameters under compressive stresses are presented in Figure 6.

Similar to the chloride profiles, *D_ac_* decreased with the applied compressive stresses initially, and then increased with an increased in stress levels compared to the specimens without stress after 2 or 3 months of ingression. For example, when the compressive stress level changed from 0 to 0.2, the value of *D_a__c_* decreased from 1.03 × 10^−11^ to 0.62 × 10^−11^ m^2^/s, reduced by about 39.5%. When the compressive stress level changed to 0.6, the value of *D_ac_* was 1.84 × 10^−11^ m^2^/s, increasing by 78.4%.

As shown clearly in Figure 6, creep has a significant effect on the chloride permeability at high stress levels. For example, at the compressive stress level of 0.7, the value of *D_ac_* increased from 1.98 × 10^−11^ to 6.13 × 10^−11^ m^2^/s when the applied time changed from 2 to 6 months; it increased more than threefold. From this experiment, it is evident that creep is a function of the damage in concrete due to nucleation and propagation of microcracks over time at high stress levels, which provides access to the chloride transport. Note in this figure that creep at low stress level had no significant effect on material damage. Therefore, the chloride concentrations and the chloride diffusion coefficients changed little over time at the low compressive level of 0.2.

**Figure 7:**
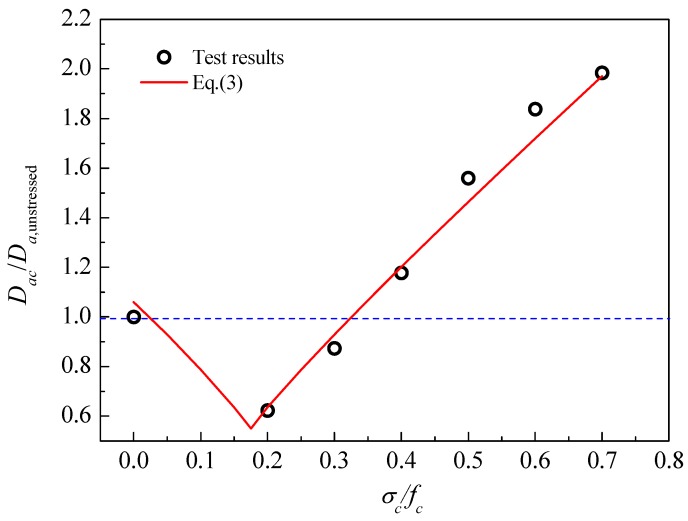
Relative chloride diffusion coefficient under compression after 2 months of exposure.

Referring to the above results, when the chloride transport is used to predict the service life of concrete structures, the stress state of the service structure should be taken into account; material tests without stress will not give an adequate assessment of the concrete. As shown in Figure 7, there is a good relationship between the compressive stress levels and the diffusion coefficients, and a threshold stress level for the chloride diffusion coefficient of compressive concrete when it was tested after two months of exposure, which is approximately 35% of the ultimate compressive strength. This is essentially the cracking of bond microcracks in the interfacial transition zone (ITZ). As shown in Figure 5, the stress-lateral strain curve slightly deviates from linearity above a stress level of 0.2 *f*_c_, indicating the onset of localized microcracking in the ITZ. However, no cracking occurs in the mortar matrix at this stage [16]. Therefore, at stress levels below this threshold value, the chloride permeability decreased firstly due to the consolidation or closing of microcracks and voids in concrete under compression (≤0.2 *f*_c_), and then increased a little due to the microcracking at the ITZ, as illustrated in Figure 7. With the increase of compressive load, the microcracks propagate stably until reaching a critical stress corresponding to the point where the volumetric strain achieves its maximum value. Thereafter, the instability of the microcracks initiates and they propagate in different directions into the matrix and coalesce as a single or several major cracks [17,18]. As a result, the chloride permeability increased rapidly at stress levels beyond the threshold stress level.

Accounting for the effect of threshold stress level, the relationship between the compressive stress and the chloride diffusivity can be predicted by Equation (3):

*D*_ac_/*D*_a,unstress_ = *A* + *B* |λ − λ_C_|*^m^* (3)

in which *D*_ac_ is the chloride diffusion coefficient of stressed concrete; *D*_a,unstress_ is the chloride diffusion coefficient of unstressed concrete; *A*, *B* and *m* are empirical coefficients; λ is the compressive stress level; λ_C_ is the threshold stress level. In this study, the values of *A*, *B* and *m* are 0.55, 2.57 and 0.92, respectively. The threshold stress level is 0.35. On the basis of these values, the chloride diffusion coefficients of concretes subjected to different compressive stress levels can be obtained. As shown in Figure 7, the proposed model fit well with the experimental results.

### 3.3. Chloride Permeability of RAC under Flexural Stress

#### 3.3.1. Chloride Profile

The chloride profiles in RACs subjected to the flexural stresses and no stress are shown in Figure 3b. The chloride content at the same depth in RAC under high flexural stress is more than that under low stress for the same exposure time. Figure 4b demonstrates the chloride concentrations at 17.5 mm depth of concrete under flexural stresses after different exposure times. It can be seen clearly from this figure that the chloride content increased more rapidly over time than the one under compressive stress.

**Figure 8:**
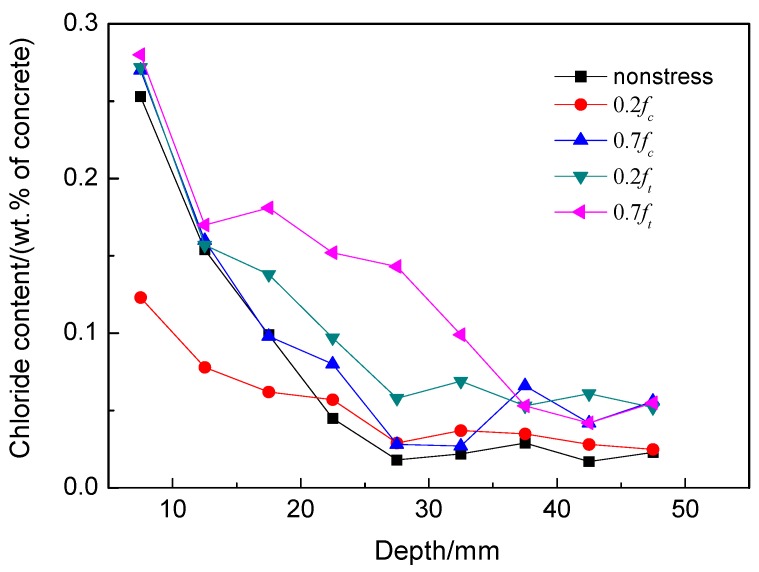
Comparison of chloride profiles under compressive and flexural stresses after 5 months’ exposure.

The comparison of chloride contents in compressive and tensile concretes as displayed in Figure 8 show that the RAC is very susceptible to the flexural or tensile stresses. Under flexural stresses, the microcracks in the ITZ tend to be open and unstable, thus increasing the connectivity of pores in the concrete and possibly accelerating the diffusion and absorption process. Although microcracks may self-heal and pozzolanic activity fills the pores and cracks gradually, the opening and propagating of microcracks predominated in RAC under flexural or tensile stress.

#### 3.3.2. Chloride Diffusion Coefficient

**Figure 9:**
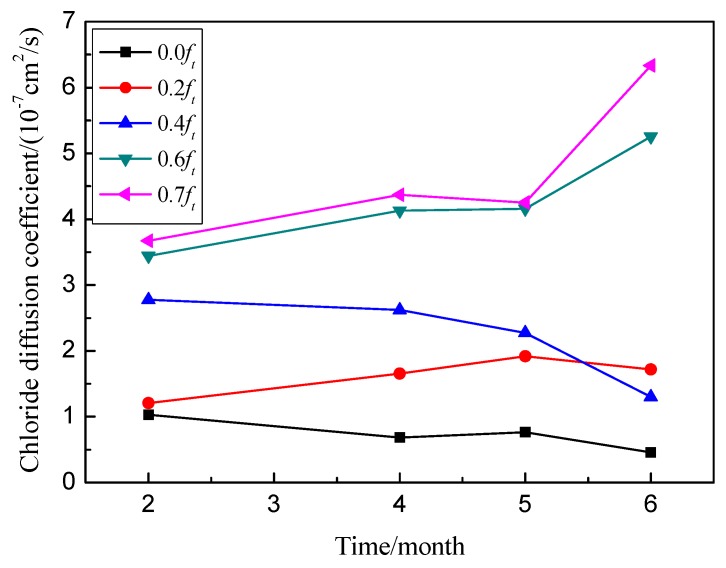
Relationship between chloride diffusion coefficients and exposure times under flexural stresses.

**Figure 10:**
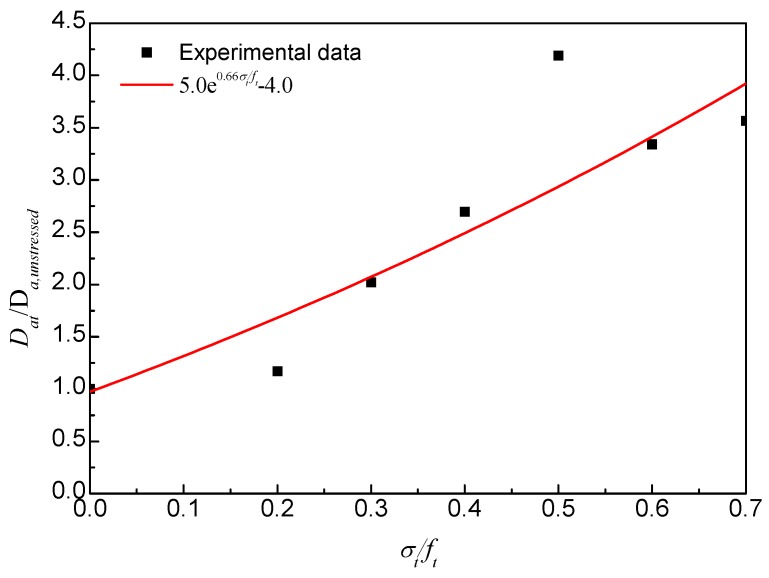
Relative chloride diffusion coefficient under flexural stresses after 2 months of exposure.

The chloride diffusion coefficients of RAC under flexural stresses (*D_at_*) are shown in Figure 9. Note that the chloride diffusion coefficients are affected by the flexural stress level. Similar to the chloride profiles, the chloride diffusion coefficient increased with the applied flexural stresses. When the flexural stress level changed from 0 to 0.2, the value of *D_at_* increased from 1.03 × 10^−11^ to 1.21 × 10^−11^ m^2^/s after 2 months of exposure, increasing by 17.5%. When the flexural stress level changed to 0.6, the value of *D_at_* is 3.44 × 10^−11^ m^2^/s increased more than threefold. As shown in Figure 10, there is a good relationship between the flexural stress levels and the diffusion coefficients, which can be described by an exponential function. Furthermore, the chloride diffusion coefficient changes with time due to the influence of creep damage, especially at high flexural stress levels.

**Figure 11:**
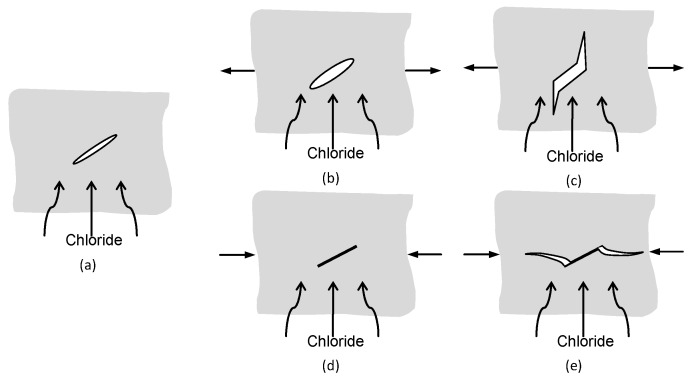
Load-induced cracking or damage in concrete: (**a**) original crack in concrete; (**b**) crack opening under lower tension; (**c**) crack propagating along the direction normal to the external tension; (**d**) crack closed under lower compression; and (**e**) crack growing toward the direction of external compression.

As shown in Figure 6 and Figure 9, the permeability of chloride under flexural stress is more sensitive than that under compressive stress. As shown in Figure 11, the damage developed differently with the geometry changes and the propagating pattern of microcracks under different external loads. As the flexural or tensile stress was applied, the microcrack in the ITZ of RAC opened but remained stable at a low stress level, which provides easy access to the chloride transport. However, the microcrack closed under compression, and the volume deformation of concrete remained in a compressive state at a same stress level, which blocks the diffusion of chloride ions. Therefore, the value of *D*_a_ increased under flexural load but decreased under compressive load at stress levels of 0.2 and 0.3. With the increase of applied external flexural load, the microcrack in the ITZ of RAC will overcome the restriction of mortar and propagate along the direction normal to the external load but parallel to the chloride diffusion. Under compressive load, however, the microcrack will grow along a curved path and eventually turn to a direction parallel to the applied load [18,19]. This propagation direction is somewhat normal to the direction of chloride transport. Thus, the microcrack length has no significant effect on the permeability of chloride under compression, and the permeability increases due to the growth of more microcracks under compressive stress. Therefore, due to the lower damage and the growing pattern of microcracks, the permeability of chloride in concrete under compressive load is much less sensitive than that under flexural load. From the growing process of microcracks and the failure mechanism of RAC, mortar cracking has a more significant effect on the chloride transport in concrete than ITZ cracking when the applied stress beyond the threshold stress level; this experiment has verified those results.

## 4. Conclusions

The permeability of a concrete with RA and supplementary cementitious materials is lower than a concrete with natural aggregates and no supplementary cementitious materials due to the on-going hydration and the pozzolanic reaction provided by the PHS and GGBS additives. The mixture of RAC in this study can be used as structural concrete.

The chloride permeability of RAC changes with the exposure time and the subjected stress level. At low compressive stress levels, the load closed some microcracks in the ITZ and micropores in cement paste, thus reducing the permeability of chloride in concrete. However, in a relatively short time the permeability of chloride increased with increasing compressive stress when it exceeded a threshold stress level of approximate 35% of the ultimate compressive strength. Under flexural stress, the permeability of chloride always increased with an increasing stress level because of the opened microcracks and pores in RAC, the cumulating damage, and the growing direction of microcracks, which was parallel to the direction of chloride diffusion.

The permeability of chloride under flexural load is much more sensitive than that under compressive load. From the cracking mechanism of microcracks in RAC under different external loads, mortar cracking has more of a significant effect on the chloride transport in concrete than cracking in the ITZ when the applied stress beyond a threshold stress level. Creep also has a significant effect on the permeability of chloride in concrete at high stress levels, contributing to damage in concrete by nucleation and propagation of microcracks over time.

## Figures and Tables

**Table 1:** Chemical composition of PHS and GGBS (mass%).

Material	CaO	SiO_2_	MgO	P_2_O_5_	Al_2_O_3_	Na_2_O	Fe_2_O_3_	F
PHS	47.91	36.81	2.56	2.73	4.47	0.49	2.27	2.65
GGBS	40.15	33.42	8.35	0	14.69	0.24	0.93	0

**Table 2:** Mix proportions of concretes.

Ingredients	RAC (Quantity: kg/m^3^)	NAC (Quantity: kg/m^3^)
Cement	276	345
Phosphorous slag	34.5	0
Ground granulated blast-furnace slag	34.5	0
5–20 mm natural aggregate	778.5	1038
5–20 mm recycled aggregate	259.5	0
Sand	677	677
FDN superplasticizer	15.33	0
Water	171	171

**Table 3:** Permeability of concrete without stress.

Permeability Index	RAC	NAC	RAC/NAC
Chloride permeability (10^–11^ m^2^/s)	1.03	2.88	35.8%
Air permeability (Ln(bar)/min)	0.0240	0.0476	50.4%

## Abbreviations

The following abbreviations are used in this manuscript:

RAC: Recycled aggregate concrete
NAC: Natural aggregate concrete
RA: Recycled-concrete aggregate
NA: Natural aggregate
PHS: Phosphorous slag
GGBS: Ground granulated blast-furnace slag
ITZ: Interfacial transition zone
